# Prebiotic and Probiotic Fortified Milk in Prevention of Morbidities among Children: Community-Based, Randomized, Double-Blind, Controlled Trial

**DOI:** 10.1371/journal.pone.0012164

**Published:** 2010-08-13

**Authors:** Sunil Sazawal, Usha Dhingra, Girish Hiremath, Archana Sarkar, Pratibha Dhingra, Arup Dutta, Priti Verma, Venugopal P. Menon, Robert E. Black

**Affiliations:** 1 Johns Hopkins Bloomberg School of Public Health, Johns Hopkins University, Baltimore, Maryland, United States of America; 2 Department of Biochemistry, Center for Micronutrient Research, Annamalai University, Chidambaram, India; Institute of Clinical Effectiveness and Health Policy, Argentina

## Abstract

**Background:**

Recent reviews suggest common infectious diseases continue to be a major cause of death among preschool children in developing countries. Identification of feasible strategies to combat this disease burden is an important public health need. We evaluated the efficacy of adding prebiotic oligosaccharide and probiotic *Bifidobacterium lactis* HN019 to milk, in preventing diarrhea, respiratory infections and severe illnesses, in children aged 1–4 years as part of a four group study design, running two studies simultaneously.

**Methods and Findings:**

In a community based double-masked, randomized controlled trial, children 1–3 years of age, willing to participate, were randomly allocated to receive either control milk (Co; n = 312) or the same milk fortified with 2.4 g/day of prebiotic oligosaccharide and 1.9×10^7^ colony forming unit (c.f.u)/day of probiotic *Bifidobacterium lactis* HN019 (PP; n = 312). Children were followed up for 1 year providing data for 1–4 years. Biweekly household surveillance was conducted to gather information on compliance and morbidity. Both study groups were comparable at baseline; compliance to intervention was similar. Overall, there was no effect of prebiotic and probiotic on diarrhea (6% reduction, 95% Confidence Interval [CI]: −1 to 12%; p = 0.08). Incidence of dysentery episodes was reduced by 21% (95% CI: 0 to 38%; p = 0.05). Incidence of pneumonia was reduced by 24% (95% CI: 0 to 42%; p = 0.05) and severe acute lower respiratory infection (ALRI) by 35% (95% CI: 0 to 58%; p = 0.05). Compared to children in Co group, children in PP group had 16% (95% CI: 5 to 26%, p = 0.004) and 5% (95% CI: 0 to 10%; p = 0.05) reduction in days with severe illness and high fever respectively.

**Conclusions/Significance:**

Milk can be a good medium for delivery of prebiotic and probiotic and resulted in significant reduction of dysentery, respiratory morbidity and febrile illness. Overall, impact of diarrhea was not significant. These findings need confirmation in other settings.

**Trial Registration:**

ClinicalTrials.gov NCT00255385

## Introduction

Globally, 5.2 million children under five years of age die every year due to preventable infectious diseases like pneumonia, diarrhea, malaria and measles [1]. Recent findings suggest 21% of global deaths and disability adjusted life years in children younger than 5 years of age are attributable to under nutrition and its synergistic relationship with preventable infectious diseases [2], [3]. Interventions are needed for prevention of these diseases for achieving Millennium Development Goals for child survival and reduction in child mortality by two-thirds by 2015 [4]–[6].

Probiotic bacteria benefit the host by adhering to the gut epithelium, stimulating host immune response, inhibiting epithelial and mucosal adherence of pathogens and producing antimicrobial substances [7]. Non-digestible carbohydrates that favor the growth and/or activity of probiotic bacteria are termed Prebiotics [8]. There has been increasing evidence in the last decade for efficacy of probiotic agents in treatment of acute diarrhea [7], [9]–[12], persistent diarrhea [13] and prevention of antibiotic associated diarrhea [14]–[16]. The evidence for impact on non-diarrheal illnesses has been unclear [17]–[20]. The data on efficacy for prevention of morbidity has been limited to small studies, mainly hospital based with short follow up or day care center based, with small sample size and short follow-ups [21]–[22]. Until date, only three randomized controlled trials have reported role of probiotics in prevention of community-acquired diarrhea [18], [20], [23]. Of these, two were in day care centers [18], [20] one of which was in developed country [20]. Data on combined use of prebiotics and probiotics in preventing common childhood illnesses in a community setting from developing countries is lacking as are data evaluating the effect on illness other than diarrhea.

We undertook a community-based, doubled-masked randomized trial with four arms to evaluate the effect of two different milk interventions in comparison to their respective control groups (essentially running two trials concurrently with a common randomization). Two groups evaluated impact of fortifying a regular milk with micronutrient bundle in comparison to same milk without fortification; and the other two groups evaluated fortification of a pre-fortified premium milk with prebiotic and probiotic in comparison to same milk without prebiotic and probiotic fortification. In this paper we are reporting the results of the two arms evaluating efficacy of consumption of prebiotic oligosaccharide and probiotic *Bifidobacterium lactis* HN019 fortified milk for a period of one year, in preventing childhood morbidity among children 1–4 years old in a peri-urban community based setting in India. The results of the other two arms are reported separately in a companion paper [24].

## Methods

The protocol for this trial and supporting CONSORT checklist are available as supporting; see Checklist S1 and Protocol S1.

### Population Description and Eligibility

The trial was carried out between April 2002 and April 2004, at Sangam Vihar, New Delhi, India. Detailed population description has previously been reported [25]. Briefly most of the inhabitants are migrants from eastern Uttar Pradesh, Bihar and Rajasthan. Literacy rates are low with 50% of the women being illiterate. About 80% men work as daily wage laborers or in factories, while 95% of women are housewives. Average family income is below 600 $/year. Community has minimal access to sewage, drinking water and paved roads. Diarrhea and respiratory illnesses are common causes of childhood mortality and morbidity. Breastfeeding is a common universal practice in the first year of life, though it starts declining after first year.

From a regularly updated database, all permanent resident families in area with children 1–3 years were invited to participate in the study and consent sought. Children with severe malnutrition needing rehabilitation by protocol were to be excluded; however, no such child was encountered. Children allergic to milk or with history of lactose intolerance were not enrolled into the trial.

### Ethics

The study protocol was approved by the human research and ethical review committees at the Johns Hopkins University, USA and the Annamalai University, India. The purpose of the study was explained to the parents in the local language, and a written informed consent was obtained. Procedure consisted of supervisor visiting the household and in presence of a third party, obtaining the consent from the mother or father after reading the consent form to them. Parents were given a choice to sign the consent form or if they were illiterate and/or could not sign, supervisor and the witness signed to document the consent.

This procedure had been approved by both institutional review boards' as majority of the mothers cannot sign in this population and taking thumb impression is stigmatized due to misuse during colonial era.

### Enrollment and baseline evaluation

Consented children were enrolled into the study and scheduled for baseline assessments in the clinic. Detailed physician examination of the child, blood sampling for assessment of body iron stores and anaemia, and weight (SECA Corporation, Columbia MD/ATCO Weighing Solutions Company Ltd, India) and height/length (Shorr Productions, Olney, MD) measurements were undertaken. Venous blood sample was collected by a trained nurse in trace free syringes to avoid micronutrient contamination. Blood samples were analyzed for a detailed hemogram using a coulter automated flow cytometer (Beckman-Coulter, Fullerton, CA), zinc protoporphyrin using hematoflourometer (AVIV Biomedical, Lakewood, NJ), serum ferritin and serum transferrin by commercial enzyme linked immunosorbant assay kits (Spectro Ferritin Kit, Spectro Transferrin kit; Ramco Laboratories, Inc, Houston, Texas). In our study, children with hemoglobin (Hb) ≤100 g/L were considered anemic and classified as iron deficient if they satisfied any two of the following four conditions: Serum ferritin ≤12 µg/L, serum transferrin >8.3 µg/ml, hematocrit ≤30%, zinc protoporphyrin ≥80 µmol per mole of heme.

### Randomization and Masking

The study was a double blind randomized controlled trial with four arms wherein we evaluated the effect of two separate interventions in comparison to their respective controls. Four letter codes namely A, B, C or D were identified for each treatment group across the two trials. Permuted fixed block randomization with block length of 16 was used.

Two separate randomization lists were generated using an in-house computerized randomization schedule - Strata one for baseline Hb>70 g/L and strata two for baseline Hb≤70 g/L. Based on their baseline Hb, children were stratified into these strata and assigned a treatment code. The supplementation sachets were identical in color, size (weight 32 g), taste, and were labeled with letter code. In the field, the letter code of the supplementation box was stripped off and labeled with child's identification information. The product corresponding with the letter code was known only to the manufacturing supervisor at Fonterra Brands (Singapore) Pte. Ltd. It was not known to investigators or anyone in the field until study completion and analysis. Morbidity impact of the micronutrient arm and its control has been previously published [25].

### Sample Size Estimation

The sample size was determined on the assumption that prebiotic and probiotic intervention would decrease diarrhea incidence by 15% and episodes of pneumonia by 25% with alpha of 0.05 and 90% power. Allowing a 10% increase in sample to account for variation in rates and 10% more for possible attrition, it was decided to enroll 312 children per group.

### Intervention

Fonterra Brands (Singapore) Pte. Ltd. provided fortified and control milk powder packed into 32 g single serve sachets. During enrollment into the study, mothers were explained the procedure to reconstitute the milk powder before feeding. Both groups received 21 sachets weekly at home by the Milk assistant (MA), with an advice to consume three sachets a day. The intervention was carried out for 1 year. Data on compliance and unused sachets were collected every week. Intervention (fortified milk per 3 serves a day) was designed to deliver 2.4 g of prebiotic oligosaccharide and 1.9×10^7^ c.f.u of probiotic *Bifidobacterium lactis* HN019. Oligosaccharide acted as a substrate, to facilitate the growth and activity of *Bifidobacterium lactis* HN019 in the gastrointestinal tract. The composition of milk in PP and Co group is given in Table 1. Irrespective of group allocation, all children with severe anemia at baseline were given a therapeutic dose of iron for 3 months in addition to their milk supplement.

**Table 1:** Composition of prebiotic and probiotic fortified milk and control milk.

Nutritive Value (per day)	Prebiotic & Probiotic fortified Milk (PP)	Control milk (CO)
Energy (kJ)	1890	1890
Protein (g)	20.1	20.1
Carbohydrates (g)	50.1	50.1
Fat (g)	19.2	19.2
Vitamin A^a^ (µg)	300	300
Vitamin D (µg)	5.1	5.1
Vitamin E^b^ (mg)	6	6
Vitamin C (mg)	48	48
Folate DFE^c^ (µg)	114	114
Vitamin B _12_(µg)	2.7	2.7
Calcium (mg)	720	720
Phosphorous (mg)	540	540
Iron (mg)	5.4	5.4
Zinc (mg)	3.3	3.3
**Prebiotic-oligosaccharides (g)**	2.4	0
***Bifidobacterium lactis*** ** HNO19** (cfu)^d^	1.9×10^7^	0

a Retinol activity equivalents.

b α-tocopherol equivalents.

c Dietary Folate Equivalents.

d Colony forming unit.

**Milk Ingredients:** Skim milk, Corn syrup solids, Cream, Sucrose, Vegetable oils (soya and sunflower), Lactose, Fish oil, Lecithin, Vanillin, **Vitamins:** Vitamin A, Vitamin D_3_, Vitamin E, Thiamin hydrochloride, Pyridoxine hydrochloride, Vitamin C, Folate, Niacinamide, **Minerals:** Ferrous sulphate, Zinc sulphate (Fortified milk contains additional prebiotic and *Bifidobacterium lactis* HNO19).

### Follow up Observation

A team of Morbidity supervisors (MS) undertook twice weekly home visits to collect prospective follow up morbidity information. Information on compliance to intervention was collected by both Milk (MA) and Morbidity (MS) teams. Before the start of the trial, we organized workshops to train and establish reliability among the field team members for measuring respiratory rate (RR), temperature and lower chest in drawing. Reliability exercises were repeated at scheduled intervals. At each home visit, morbidity information for each of the previous 3–4 days since last visit was recorded, including number of diarrheal stools, consistency of stools and blood in stools, pneumonia, fever, ear discharge, measles, vomiting and feeding history. During these biweekly visits, MS team measured RR and temperature of the child, and looked for signs of lower chest in-drawing. Whenever any of these parameters was found to be more than the normal range, children were referred to study physician for further examination.

Household was revisited by MS/MA on the next day, in case the child or the parent was not available on a scheduled visit day. Two levels of supervision and random checking were established above the MA and MS level to ensure quality control of data. Mothers were advised to contact study physicians at the clinic if they felt that the child was sick between visits. Treatment of diarrhea, dysentery and pneumonia as per WHO guidelines was provided free to the participating children throughout the study. All visits either to the study physicians or to private physicians were recorded.

The anthropometric measurements were repeated after 6 months and one year of intervention. The blood sampling was repeated after one year of supplementation.

### Definitions of Outcomes

Primary outcomes were not explicitly prespecified in the protocol; the intent was to evaluate impact on common childhood illnesses including diarrhea, pneumonia, and febrile illness. However, the sample size was estimated based upon the effects on diarrhea and pneumonia.

Diarrhea was defined as ≥3 loose or watery stools in 24 hours, and diarrheal episodes were considered recovered on first day of three diarrhea free days. Dysentery was defined as diarrhea with visible blood in stools. We used field based pneumonia definition [26]. Severe ALRI was defined as RR> = 50/min, Pneumonia was defined if a) severe ALRI was present or b) RR was> = 40/min but was accompanied by either lower chest in-drawing or temperature of > = 37.7°C.

Axillary temperature of ≥38.4°C was considered as high fever. Severe illness was defined as days with temperature ≥38.4°C or hospitalization or RR≥50/min or chest in-drawing associated with it.

### Data Management and Statistical Analysis

The data collected in the field on a pre-designed data collection form was entered, collated and stored in the relational database management system developed in Oracle 8i with stringent range, consistency and logical checks. Real time data entry, data being entered by the end of next day after data collection, ensured data quality and accuracy. A double data entry and manual checking of frequencies was performed during data cleaning. We performed intent to treat analysis, i.e. all children were included in analyses irrespective of supplement adherence. For children out-migrating or withdrawing from the study, data were included until the date of censorship. Person-time analysis was performed with actual follow-up as denominator. For the effect on incidence (diarrhea, ALRI, dysentery, measles), relative risk has been estimated using Poisson regression and for prevalence, odds ratio has been estimated using General Linear Model for binomial outcomes (maximum likelihood logit estimation for grouped data). Both estimations were performed in STATA 9.2, (Stata Corp, Union Station, TX, USA), and SPSS ver. 12.0 (SPSS Inc., NJ, USA). Anthropometric Z scores were calculated using WHO standards [27].

## Results

**Figure 1:**
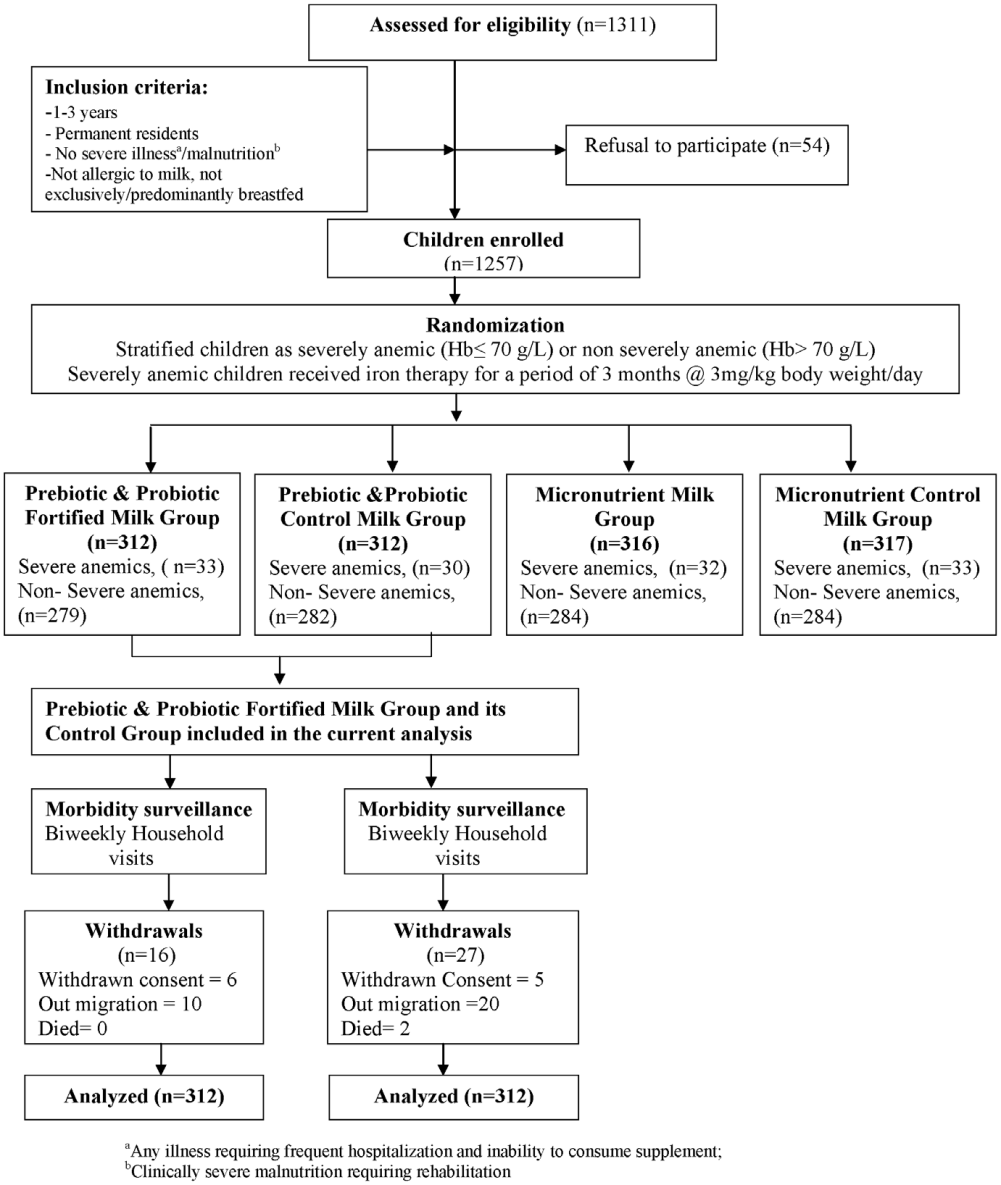
Schematic representation of trial design.

Out of 651 eligible children contacted, 624 children [312 in intervention (prebiotic and probiotic milk (PP), 312 in control milk (Co)] were enrolled into the trial (Figure 1). At enrollment, children allocated to the groups were comparable for socio economic, demographic descriptors, hematology and nutritional status (Table 2). At baseline, 55% of children in both the groups were partially breastfed. The adherence to study milk feeds was high and similar in both groups, 84.0% children in the PP group and 82.7% in Co group consumed two or three servings on >80% days. This did not vary by the intervention period. No adverse event because of intervention was observed during the course of the study. Of the total follow up period, information was not available for 19% of the child-days in the prebiotic and probiotic group and 21% of the child days in the control group due to non-availability of the children and their parents. Six children in the prebiotic and probiotic group and five children in the control group had withdrawn consent during the follow up.

**Table 2:** Baseline characteristics of prebiotic and probiotic fortified and control milk groups.

	PP Group (n = 312)	Co Group (n = 312)
**Age** (mo)^a^	22.2±6.4	22.9±6.8
	(21.7)^b^	(21.6)
Age (≤24 months)^c^	191 (61.2)	191 (60.9)
Illiterate father^c^	43 (13.8)	54 (17.3)
Illiterate mother^c^	152 (48.7)	155 (49.7)
**Occupation father**		
Daily wage labor^c^	105 (33.7)	112 (35.9)
**Occupation mother**		
Housewives^c^	299 (95.8)	301 (96.5)
Socio economic status score^a^	7.66±2.57	7.10±2.45
**Water supply**		
Tap water^c^	189 (60.6)	195 (62.5)
**Hematological Status**		
Hemoglobin^a^^,^ d (g/L)	91.1±15.6	91.0±14.9
	93.0 (61.0;114.0)^e^	92.0 (63.0; 114.0)
Zinc protoporphyrin^a^^,^ d (µmol/mole heme)	193.46±125.65	199.12±124.99
	151.5 (42.0; 481.7)^e^	167 (55.8; 456.6)
Serum transferrin^a^^,^ d (g/L)	15.25±8.85	15.21±8.79
	12.97 (5.68; 35.70)^e^	13.22 (5.22; 35.49)
Serum ferritin^a^^,^ d (µg/L)	9.23±7.96	9.87±9.09
	6.81 (2.29; 25.7)^e^	6.75 (2.15;29.76)
Redcell distribution width^a^^,^ d (%)	19.36±2.75	19.35±2.68
	19.1 (15.1; 24.1)^e^	19.3 (14.9; 23.8)
Iron deficient anemic^c^	158 (54.1)	168 (56.9)
**Nutritional status**		
Normal^c^	107 (34.3)	95 (30.4)
Wasted and Stunted^c^	53 (17.0)	46 (14.7)
Wasted^c^	15 (4.8)	14 (4.5)
Stunted^c^	137 (43.9)	157(50.3)

a Mean±SD.

b Median age.

c Number (%).

d Reference values for hematological markers: Hb>100 g/L, Zinc Protoporphyrin <80 µmol per mole of heme, serum Transferrin ≤8.3 µg/ml, serum Ferritin >12µg/L, Red Cell Distribution Width ≤14%.

e Median (5^th^; 95^th^ percentile).

Overall, children in the PP group had 6% lower rate of diarrhea (95% CI: −1 to 12%; p = 0.08) compared to children in Co group. Ancillary analysis based on age revealed a significant age interaction, therefore making overall rate reduction less meaningful; for children aged 12 to 24 months rate of diarrhea was [1% lower (95% CI: −11% to 11%), p = 0.91] and children aged >24 months it was [10% lower (95% CI: 2% to 17%), p = 0.02]; p-value for test of difference = 0.03. The incidence of dysentery was 21% lower (95% CI: 0 to 38%; p = 0.05) in the prebiotic and probiotic group than the control group (Table 3).

**Table 3:** Effect of prebiotic oligosaccharide and probiotic *Bifidobacterium lactis HN019* fortified milk on common childhood morbidities.

	PP group (n = 312)		Co group (n = 312)		Odds Ratio (95% CI)	p value
	Actual numbers	Episodes per child year	Actual numbers	Episodes per child year		
**Gastrointestinal morbidity**						
Diarrhea episodes (1–4 y)	1641^a^	6.21^b^	1697^a^	6.61^b^	0.94 (0.88–1.01)	0.08
≤24 mo	603	2.3	563	2.2	0.99 (0.89–1.11)	0.91
>24 mo	1038	3.92	1134	4.41	0.90 (0.83–0.98)	0.02
Dysentery episodes	125	0.47	154	0.6	0.79 (0.62–1.00)	0.05
**Respiratory morbidity**						
Pneumonia episodes^c^	90	0.34	115	0.45	0.76 (0.58–1.00)	0.05
Severe ALRI episodes^d^	34	0.13	51	0.20	0.65 (0.42–1.00)	0.05
**Febrile illness and others**						
Days with severe illness (1–4 y)	473	1.8	550	2.14	0.84 (0.74–0.95)	0.004
≤24 mo	153	0.58	177	0.69	0.80 (0.65–0.99)	0.05
>24 mo	320	1.21	373	1.5	0.85 (0.73–0.98)	0.03
Days with ear discharge	1550	5.87	1613	6.3	0.93 (0.87–1.00)	0.06
Days with high fever	2798	10.6	2865	11.2	0.95 (0.90–1.00)	0.05
Measles	5	0.02	10	0.04	0.49 (0.17–1.42)	0.19
Doses of antibiotics consumed	7402	28.02	7625	29.7	0.94 (0.91–0.97)	<0.001

a Actual numbers.

b Episodes per child year.

c Field based Pneumonia Definition a) If RR was > = 50/min, or b) RR was > = 40/min but was accompanied by lower chest in-drawing or temperature of ≥37.7°C.

d RR> = 50/min.

There was a 24% reduction (95% CI: 0 to 42%) in incidence of pneumonia (field based definition) and 35% reduction (95% CI: 0 to 58%) in severe ALRI in the prebiotic and probiotic fortified group compared to the control group. Although statistically significant, the confidence interval of this difference was wide and compatible with small or no difference at upper bound of confidence interval (Table 3).

The prevalence of severe illness among children consuming prebiotic and prebiotics-fortified milk was 16% (95% CI: 5 to 26%; p = 0.004) lower than the control group. This was similar among children 1 to 2 years [20% lower (95% CI: 1 to 35%); p = 0.05], and 2 to 4 years [15% lower (95% CI: 2 to 27%); p = 0.03] (Table 3). Children in the prebiotic and probiotic fortified group showed a statistically significant 5% fewer days with high fever and 7% lower prevalence of ear discharge compared to control group. The antibiotics usage was [6% (95% CI: 3 to 9%), p<0.001] less among children consuming fortified milk (Table 3).

Sub group analyses based on breast-feeding, malnutrition and anemia have been presented in supplementary tables (see Tables S1, S2, S3, S4, S5 and S6).

## Discussion

This study, reports the first large randomized controlled trial, evaluating effect of providing combination of prebiotic and probiotic in milk for one year on both gut and non-gut related illnesses among children in a community based setting. We found a significant beneficial effect on dysentery, pneumonia and febrile illnesses. Effect on diarrhea was restricted to children aged >24 months. Although age interaction was statistically robust, given the exploratory nature of this finding, results need to be interpreted with caution.

Health effects of probiotics can vary by the specific probiotic used. *Bifidobacterium lactis* HN019 used in this study has shown extensive safety and immune-stimulant activity in animal models including impact in animal models for *E.coli* and rotavirus [28]–[30]. Immune-stimulant activity among healthy adult volunteers, with no notable adverse health events has been documented [31]–[33].

Both intervention and control group children consumed similar quantity of milk, and milk in both groups was iso-energic, with identical macronutrient quality and quantity as well as quantity of vitamins and minerals. The only difference was the milk for children allocated to the prebiotic and probiotic group delivered additionally 2.4 g/day of prebiotic oligosaccharide and 1.9×10^7^ c.f.u/day of probiotic *Bifidobacterium lactis* HN019. The results in this study need to be interpreted as the effects of the combination of oligosaccharide and *Bifidobacterium lactis* HN019. In addition, in the interpretation of results we need to consider that the base milk used was fortified with iron, zinc and vitamins and improved in nutrient composition (Table 1). Although unlikely but, we cannot exclude the possibility that this is important for success of intervention and therefore effects observed may not be same if prebiotic and probiotic were added to regular unfortified milk.

Enrolled children were randomly allocated to the two intervention groups, and the participants, health workers and investigators were masked to group allocation. The similar pattern of compliance between intervention and control groups further supports the belief that masking was very good. As the study relied on active, biweekly follow up by household based surveillance, this would have identified the occurrence of almost all the clinical outcomes of interest, thereby further limiting the possibility of a reporting bias.

Although there is substantial evidence for the role of probiotics in diarrhea, majority of that evidence is either from treatment of acute diarrhea [7], [9], [10], [12], persistent diarrhea [13] or antibiotic associated diarrhea [14]–[16] and occurrence of nosocomial infections [34]–[36]. This evidence cannot be extrapolated to prevention of diarrhea in healthy children. The overall reduction in incidence of diarrhea of 6% in this study is similar to, the only other large community based trial, conducted among Peruvian infants and young children [23]. The results of a significant reduction in dysentery episodes, and diarrheal episodes in children 2–4 years is consistent with results from other published randomized controlled trials evaluating prevention of acute diarrhea acquired in day care centers [20], [37]–[41]. The significant differential effect of probiotic on the incidence of diarrhea among younger and older children may explain variations in the results of the previous studies. The lack of effect in children below 24 months of age could be due to one or more of the following: a) difference in the constitution of the gut flora among children >24 months of age, b) a shift in Th1 and Th2 balance. During infancy, the cellular immune system is maturing with a shift from Th2 predomination at birth to Th1 predomination by second year of life. The effect of probiotic may be limited in infants due to intrinsic limitations in the capacity of infants to produce interferon and other Th1 interleukins (IL-2, IL12), c) Breastfeeding among younger children may have modified the effect of probiotic, as studies have suggested greater benefits of prebiotic and probiotic in non-breastfed children as compared to breast fed children [23]. We did not observe significant difference in diarrhea reduction between breast fed and non breast fed children in the age group of 12–24 months; however given lack of power we also cannot exclude it.

Although we did not evaluate etiology of diarrhea in this study, previous evaluation have shown rotavirus to be responsible for 2.3% of cases, enterotoxigenic *Escherichia coli* and enteropathogenic *Escherichia coli* as major causes with 13.5% and 6.3% of cases, while for dysentery *Salmonella species* and *Shigella species* account for 3.2 and 1.8% of cases [42]. Cholera is not endemic in this population.

A reduction in incidence and prevalence of febrile illness, pneumonia, severe ALRI, marginal reduction in ear infections and requirement for antibiotics is consistent with findings from the only three studies which have evaluated prevention of similar morbidity syndromes in healthy children [18]–[20]. However, these studies, for many of these outcomes documented trends only, due to lack of statistical power. This study which is the largest reported thus far, implemented an active home based surveillance for morbidity and had a follow up of full one year which potentially provided sensitive estimation of the morbidity. The beneficial effects documented in this study are multi-systemic, indicating that the underlying mechanism for the beneficial effects most likely was due to improved immune response to viral and bacterial infections. Improvement in immunity could have been mediated through improved production of antimicrobial substances, attachment in intestinal mucosal sites, inhibition of the attachment and growth of pathogenic organisms by achieving competitive exclusion and microbial balance leading to regeneration of gut epithelium and consequently resulting in better absorption of nutrients [43]–[46]. However given the variation in effects of probiotics upon such immune mechanisms, the observed effects should be interpreted as effects of the preparation used in this study and not generalized to all prebiotic and probiotic combinations.

Reducing the preventable childhood illnesses among preschool children in developing countries is an important public health goal, that would not only impact mortality by breaking malnutrition cycle but would also impact better development of children. The findings of this study suggest that fortification with prebiotic and probiotic together may provide one of the potential interventions to reduce the burden of common childhood morbidities. However, before any public health recommendations are made, these results need to be confirmed in varied settings and with locally available probiotic strains.

## Supporting Information

Table S1 Episodes of common childhood morbidities for children who were breast fed.
(0.04 MB DOC)

Table S2 Episodes of common childhood morbidities for children who were non breast fed.
(0.04 MB DOC)

Table S3 Effect of prebiotic oligosaccharide and probiotic Bifidobacterium lactis HN019 and fortified milk on common childhood morbidities (among anemic children).
(0.04 MB DOC)

Table S4 Effect of prebiotic oligosaccharide and probiotic Bifidobacterium lactis HN019 and fortified milk on common childhood morbidities (among non anemic children).
(0.04 MB DOC)

Table S5 Effect of prebiotic oligosaccharide and probiotic Bifidobacterium lactis HN019 and fortified milk on common childhood morbidities (among malnourished children).
(0.04 MB DOC)

Table S6 Effect of prebiotic oligosaccharide and probiotic Bifidobacterium lactis HN019 and fortified milk on common childhood morbidities (among non malnourished children).
(0.04 MB DOC)

Checklist S1 CONSORT Statement 2001 - Checklist.
(0.06 MB DOC)

Protocol S1 Protocol for Efficacy Study of Milk Fortified with Bifidobacillus lactis HNO19 and Oligosaccharides or Zinc and Iron and other Micronutrients.
(0.27 MB DOC)
