# Supplementary material for: Prebiotic and Probiotic Fortified Milk in Prevention of Morbidities among Children: Community-Based, Randomized, Double-Blind, Controlled Trial
Source: PLoS One. 2010 Aug 13;5(8):e12164. doi: 10.1371/journal.pone.0012164 (PMC2921405; doi:10.1371/journal.pone.0012164)
Supplement: Table S1 — Episodes of common childhood morbidities for children who were breast fed. (0.04 MB DOC) [file pone.0012164.s001.doc]

| **Table S1. Episodes of common childhood morbidities for children who were breast fed** | | | | |
| --- | --- | --- | --- | --- |
|  | **PP group**  **(n=171)** | **Co group (n=169)** | **OR (95% CI)** | **p value** |
| **Gastrointestinal morbidity**  Diarrhea episodes (1-4 y) | 861 | 944 | 0.89 (0.81-0.97) | 0.01 |
| ≤ 24 mo | 420 | 398 | 1.02 (0.89-1.17) | 0.79 |
| > 24 mo | 441 | 546 | 0.79 (0.70-0.90) | <0.001 |
| Dysentery episodes | 70 | 96 | 0.71 (0.52-0.97) | 0.03 |
| **Respiratory morbidity** |  |  |  |  |
| Pneumonia episodes**^a^** | 56 | 64 | 0.85 (0.60-1.22) | 0.38 |
| Severe ALRI episodes**^b^** | 23 | 33 | 0.68 (0.40-1.16) | 0.15 |
| **Febrile illness and others** |  |  |  |  |
| Days with severe illness (1-4 y) | 299 | 321 | 0.91 (0.77-1.06) | 0.23 |
| ≤ 24 mo | 129 | 144 | 0.86 (0.68-1.10) | 0.23 |
| > 24 mo | 170 | 177 | 0.94 (0.76-1.16) | 0.57 |
