# Supplementary material for: Prebiotic and Probiotic Fortified Milk in Prevention of Morbidities among Children: Community-Based, Randomized, Double-Blind, Controlled Trial
Source: PLoS One. 2010 Aug 13;5(8):e12164. doi: 10.1371/journal.pone.0012164 (PMC2921405; doi:10.1371/journal.pone.0012164)
Supplement: Table S2 — Episodes of common childhood morbidities for children who were non breast fed. (0.04 MB DOC) [file pone.0012164.s002.doc]

| **Table S2. Episodes of common childhood morbidities for children who were non breast fed** | | | | |
| --- | --- | --- | --- | --- |
|  | **PP group**  **(n=141)** | **Co group (n=143)** | **OR (95% CI)** | **p value** |
| **Gastrointestinal morbidity**  Diarrhea episodes (1-4 y) | 780 | 753 | 1.00 (0.91-1.11) | 0.93 |
| ≤ 24 mo | 183 | 165 | 0.89 (0.72-1.09) | 0.28 |
| > 24 mo | 597 | 588 | 1.01 (0.90-1.13) | 0.22 |
| Dysentery episodes | 55 | 58 | 0.91 (0.63-1.33) | 0.65 |
| **Respiratory morbidity** |  |  |  |  |
| Pneumonia episodes**^a^** | 34 | 51 | 0.65 (0.42-1.00) | 0.05 |
| Severe ALRI episodes**^b^** | 11 | 18 | 0.59 (0.28-1.26) | 0.17 |
| **Febrile illness and others** |  |  |  |  |
| Days with severe illness (1-4 y) | 174 | 229 | 0.73 (0.60-0.89) | 0.002 |
| ≤ 24 mo | 24 | 33 | 0.58 (0.34-0.98) | 0.45 |
| > 24 mo | 150 | 196 | 0.76 (0.61-0.94) | 0.01 |
