# Supplementary material for: Prebiotic and Probiotic Fortified Milk in Prevention of Morbidities among Children: Community-Based, Randomized, Double-Blind, Controlled Trial
Source: PLoS One. 2010 Aug 13;5(8):e12164. doi: 10.1371/journal.pone.0012164 (PMC2921405; doi:10.1371/journal.pone.0012164)
Supplement: Table S3 — Effect of prebiotic oligosaccharide and probiotic Bifidobacterium lactis HN019 and fortified milk on common childhood morbidities (among anemic children). (0.04 MB DOC) [file pone.0012164.s003.doc]

**Table S3: Effect of prebiotic oligosaccharide and probiotic *Bifidobacterium lactis HN019* and fortified milk on common childhood morbidities (among anemic children)**

|  | **PP group**  **(n=222)** | |  | **Co group**  **(n=228)** | **OR (95% CI)** | **p value** |
| --- | --- | --- | --- | --- | --- | --- |
| **Gastrointestinal morbidity** | |  |  |  |  |  |
| Diarrhea episodes (1-4 y) | | 1176 |  | 1191 | 0.98 (0.90-1.06) | 0.62 |
| Dysentery episodes | | 90 |  | 113 | 0.79 (0.60-1.04) | 0.10 |
| **Respiratory morbidity** | |  |  |  |  |  |
| Pneumonia episodes^c^ | | 61 |  | 90 | 0.67 (0.49-0.93) | 0.02 |
|  | |  |  |  |  |  |
| Severe ALRI episodes^d^ | | 21 |  | 43 | 0.48 (0.29-0.82) | 0.007 |
| **Febrile illness and others** | |  |  |  |  |  |
| Days with severe illness (1-4 y) | | 356 |  | 415 | 0.85 (0.74-0.98) | 0.03 |
