# Supplementary material for: Prebiotic and Probiotic Fortified Milk in Prevention of Morbidities among Children: Community-Based, Randomized, Double-Blind, Controlled Trial
Source: PLoS One. 2010 Aug 13;5(8):e12164. doi: 10.1371/journal.pone.0012164 (PMC2921405; doi:10.1371/journal.pone.0012164)
Supplement: Table S4 — Effect of prebiotic oligosaccharide and probiotic Bifidobacterium lactis HN019 and fortified milk on common childhood morbidities (among non anemic children). (0.04 MB DOC) [file pone.0012164.s004.doc]

**Table S4: Effect of prebiotic oligosaccharide and probiotic *Bifidobacterium lactis HN019* and fortified milk on common childhood morbidities (among non anemic children)**

|  | **PP group**  **(n=87)** | |  | **Co group**  **(n=82)** | **OR (95% CI)** | **p value** |
| --- | --- | --- | --- | --- | --- | --- |
| **Gastrointestinal morbidity** | |  |  |  |  |  |
| Diarrhea episodes (1-4 y) | | 433 |  | 492 | 0.83 (0.73-0.05) | 0.005 |
| Dysentery episodes | | 33 |  | 40 | 0.78 (0.49-1.23) | 0.29 |
| **Respiratory morbidity** | |  |  |  |  |  |
| Pneumonia episodes^c^ | | 29 |  | 24 | 1.14 (0.66-1.96) | 0.63 |
|  | |  |  |  |  |  |
| Severe ALRI episodes^d^ | | 13 |  | 8 | 1.53 (0.64-3.70) | 0.34 |
| **Febrile illness and others** | |  |  |  |  |  |
| Days with severe illness (1-4 y) | | 115 |  | 130 | 0.83 (0.65-1.07) | 0.16 |
