# Supplementary material for: Prebiotic and Probiotic Fortified Milk in Prevention of Morbidities among Children: Community-Based, Randomized, Double-Blind, Controlled Trial
Source: PLoS One. 2010 Aug 13;5(8):e12164. doi: 10.1371/journal.pone.0012164 (PMC2921405; doi:10.1371/journal.pone.0012164)
Supplement: Table S5 — Effect of prebiotic oligosaccharide and probiotic Bifidobacterium lactis HN019 and fortified milk on common childhood morbidities (among malnourished children). (0.04 MB DOC) [file pone.0012164.s005.doc]

**Table S5: Effect of prebiotic oligosaccharide and probiotic *Bifidobacterium lactis HN019* and fortified milk on common childhood morbidities (among malnourished children)**

|  | **PP group**  **(n=205)** | |  | **Co group**  **(n=217)** | **OR (95% CI)** | **p value** |
| --- | --- | --- | --- | --- | --- | --- |
| **Gastrointestinal morbidity** | |  |  |  |  |  |
| Diarrhea episodes (1-4 y) | | 1106 |  | 1271 | 0.88 (0.81-0.96) | 0.002 |
| Dysentery episodes | | 84 |  | 116 | 0.73 (0.55-0.97) | 0.03 |
| **Respiratory morbidity** | |  |  |  |  |  |
| Pneumonia episodes^c^ | | 70 |  | 97 | 0.73 (0.54-0.99) | 0.05 |
| Severe ALRI episodes^d^ | | 28 |  | 43 | 0.66 (0.41-1.06) | 0.09 |
| **Febrile illness and others** | |  |  |  |  |  |
| Days with severe illness (1-4 y) | | 339 |  | 427 | 0.80 (0.70-0.93) | 0.003 |
