# Supplementary material for: Prebiotic and Probiotic Fortified Milk in Prevention of Morbidities among Children: Community-Based, Randomized, Double-Blind, Controlled Trial
Source: PLoS One. 2010 Aug 13;5(8):e12164. doi: 10.1371/journal.pone.0012164 (PMC2921405; doi:10.1371/journal.pone.0012164)
Supplement: Table S6 — Effect of prebiotic oligosaccharide and probiotic Bifidobacterium lactis HN019 and fortified milk on common childhood morbidities (among non malnourished children). (0.04 MB DOC) [file pone.0012164.s006.doc]

**Table S6: Effect of prebiotic oligosaccharide and probiotic *Bifidobacterium lactis HN019* and fortified milk on common childhood morbidities (among non malnourished children)**

|  | **PP group**  **(n=107)** | |  | **Co group**  **(n=95)** | **OR (95% CI)** | **p value** |
| --- | --- | --- | --- | --- | --- | --- |
| **Gastrointestinal morbidity** | |  |  |  |  |  |
| Diarrhea episodes (1-4 y) | | 535 |  | 426 | 1.12 (0.99-1.27) | 0.08 |
| Dysentery episodes | | 41 |  | 38 | 0.96 (0.62-1.50) | 0.87 |
| **Respiratory morbidity** | |  |  |  |  |  |
| Pneumonia episodes^c^ | | 20 |  | 18 | 0.99 (0.53-1.88) | 0.98 |
|  | |  |  |  |  |  |
| Severe ALRI episodes^d^ | | 6 |  | 8 | 0.67 (0.24-1.93) | 0.46 |
| **Febrile illness and others** | |  |  |  |  |  |
| Days with severe illness (1-4 y) | | 134 |  | 123 | 0.97 (0.76-1.24) | 0.83 |
