# Supplementary material for: Prebiotic and Probiotic Fortified Milk in Prevention of Morbidities among Children: Community-Based, Randomized, Double-Blind, Controlled Trial
Source: PLoS One. 2010 Aug 13;5(8):e12164. doi: 10.1371/journal.pone.0012164 (PMC2921405; doi:10.1371/journal.pone.0012164)
Supplement: Protocol S1 — Protocol for Efficacy Study of Milk Fortified with Bifidobacillus lactis HNO19 and Oligosaccharides or Zinc and Iron and other Micronutrients. (0.27 MB DOC) [file pone.0012164.s008.doc]

**Efficacy Study of Milk Fortified with Bifidobacillus lactis HNO19 and Oligosaccharides or Zinc and Iron and other Micronutrients**

**Working Protocol**

| **Flow Chart for the Efficacy Study Design of Milk fortified with HNO19 or Iron and Zinc** |  |  |  |  |
| --- | --- | --- | --- | --- |

**Baseline Population Database**

CMR has conducted detailed survey in Sangam Vihar, which includes updated mapping and numbering of houses, census of children by age, household information and SES features. This population is being maintained under fortnightly surveillance

**Enrollment of Eligible Children**

From the available database, all eligible children between 1-3 yr. of life will be visited, consent for participation sought after evaluating inclusion and exclusion criteria, eligible children who’s parents consent to participate will be enrolled, given a study identification number which will also be used for randomization and baseline assessment forms will be filled.

**Preparatory Phase**

Pre-Randomization

**Randomization into four groups**

**Baseline Assessment**

One year follow up

**During**

**Follow up**

**Baseline Information**

- Family information – socioeconomic information, retrospective morbidity assessment for 2 weeks, with special emphasis on diarrhea and pneumonia/respiratory infection, record of dietary habits using 24 Hr dietary recall and semi-quantitative food frequency questionnaire and hygiene.
- Clinical assessment of the child will be made by the research physician and hospitalization episodes in last one month with course for each recorded.
- Blood sample for zinc and iron status.
- Baseline anthropometric measurement weight and length/height will be recorded.
- Global assessment of cognitive and motor development will be measured by the Bayley Scales of Infant Development (Ver. II).
- Parent Child Early Relationship Assessment,(PCERA), Social Referencing (SR), Activity Assessment on a sub- sample, BRIEF , HOME, Language questionnaire

Milk fortified with Bifidobacillus lactis HNO19 and Oligosaccharides

Control Formulation (Same milk without pre and probiotics)

Control Formulation (Same milk without micronutrients)

Milk fortified with micronutrients (Zinc and Iron)

**During**

**Baseline**

###### **Follow up and End Study**

###### **Weekly visitation at home by Compliance Assessment Monitor (Junior Field Worker)**

- Deliver one packet containing supplementation (21 sachets x 32g of milk powder) of the code to which the child belongs. This will be a week’s supply.
- Demonstrate the reconstitution procedure and educate the mother of dosage and frequency of administration [32 mg milk powder (1 sachet) reconstituted to 200ml and given three times a day].
- Assess and record compliance for previous week.
- Record acceptability and problems reported.

**Twice Weekly Visitation at home by Health Assessment Monitor (Senior Field Worker)** Record information on hospitalization, diarrhea, acute respiratory infection pneumonia and fever on that day and the preceding 3/4days on a standard form. This visitation form also collects data on any other illness, feeding characteristics, reported intake of milk, use of any other medication and supplements.

- Monthly information on Dietary intake using Semi-quantitative food questionnaire and 24 hr Dietary Recall at 12 months
- Blood sample collection for zinc, iron, copper and lead status at 12 months.
- The follow-up weight, height/length will be taken at 6 and 12 months of enrollment.
- Follow up Global assessment of cognitive and motor development will be measured by the Bayley Scales of Infant Development, Ver.2 at 6months and at 12months of study. After each administration of the Bayley Scales, the examiner will complete the Behavior Rating Scale (BRS) from the Bayley Scales, based on the child’s response to the structure of the testing situation.
- Parent Child Early Relationship Assessment,(PCERA), Social Referencing (SR), Activity Assessment on a sub- sample, BRIEF , HOME, Language questionnaire, Parental Stress Index (PSI), Behavior and Social Interactions (BASIS)

Control Formulation (Same milk without micronutrients

# B. THE PROJECT

## **1. Project Background and Significance**

The 1993 World Bank World Development Report (1) delivered a strong but sad reminder of the dramatic scale of suffering and death among preschool children attributable to pneumonia (2.7million in 1990) and diarrhea (another 2.5 million deaths). In terms of “disability adjusted life years” (DALYS) lost, in 0-4 years old children, respiratory infections account for 18.5% and 17.6% (in boys & girls respectively) and diarrhea accounts of another 16.2% and 15.7% respectively. The development of effective interventions to reduce morbidity from these illnesses has become a humanitarian, economic and political imperative and essential in achieving the morbidity and mortality reduction goals that the “World Summit for Children” set a decade ago. Therapy with Oral Rehydration Solution (ORS) for diarrhea, a cornerstone of the universal strategy for child survival, has low compliance due to having no effect on duration of diarrhea and has mixed impact on child mortality (2-5). Present strategies for containing morbidity and mortality due to respiratory infection focuses primarily on antibiotic treatment. Increasing prevalence of antibiotic resistant strains, poses a problem with the current strategy. Lack of any truly effective and affordable preventive vaccine, for both diarrhea and pneumonia has aroused considerable interest in food based preventive interventions. In the recent years increasing recognition and evidence regarding the role of Probiotics, and micronutrients like Zinc, as both preventive and therapeutic agents against infections has made them a potential and promising candidate.

**1.1 Role of Probiotics**

In great numbers and diversity, the microbes inhabit the intestinal tract, skin, urogenital tract, oral and nasal cavities. Hundreds of species has been identified as human commensals. They interfere with virulence of invading pathogens by what is attributed as “competitive colonization”, and poor priming of immune system (6). On rare occasions, microbes develop a pathogenic relationship with the host. It is the recognition of the effects of colonizing microbes in association with the human body, and the combination of wanting to encourage the positive and discourage the negative activities of the commensals and invading microbes that have led to the probiotic theory (7). Probiotics have been defined as live microorganisms that confer a health effect on the host when consumed in adequate amount (8). Probiotics have been suggested to play a role in a variety of health effects and mechanisms proposed for mediating these effects are numerous. These include antimicrobial activity, colonization resistance, immune effects, adjuvant effect, cytokine expression, stimulation of phagocytosis by peripheral blood leucocytes, secretory IgA, antimutagenic effects, anti genotoxic effect, influence on enzyme activity and enzyme delivery (7).

Currently Lactobacillus sp. & Bifidobacterium sp. are the best studied probiotics (9)and species of the genus Bifidobacterium are the most numerous colonizers of the intestinal tract of infants, particularly those who are breast-fed. Lactic acid bacteria (LAB) are the normal inhabitants of the human gut microflora. Recent scientific evidence indicates that dietary consumption of dairy LAB can promote enhancement of natural immunity. Certain strains of Lactobacilli and Bifidobacillus are able to enhance immunity and mediate protection against bacterial & viral infection more strongly (10). Saavedra et al. has reported that milk-based diets containing Bifidobacillus bifidum reduce the incidence and severity of diarrheal disease among infants (age 5-24months) and reduce Rotavirus infection (11). The ability of prebiotics like Oligosaccharides contained in human breast milk (12) and derived from bovine milk through hydrolysis of lactose to promote the growth of friendly LABS (unpublished observation) and preventing infection of the gut in humans has also been demonstrated. In addition there is evidence that milk-derived Oligosaccharides directly prevent infection of the gut by pathogenic bacteria, by preventing pathogen attachment to the intestinal mucosa. Recent research by the Milk and Health Research Centre has identified a new strain of Bifidobacterium lactis (B.lactis HNO19) which is particularly efficient in promoting natural immunity in humans (13)14) as well as laboratory animals (15). Dietary consumption of this bacterium has also been shown to enhance natural immunity in mice and to protect them against infection with Salmonella (16). Given that both ETEC and Enetero-aggregative E.coli which are the largest etiological agent groups in community acquired diarrhea could be affected by such a mechanism, as they both require attachment to enterocytes as a part of etiopathogenesis.

Bacteria, usually Streptococcus pneumonia or Haemophilus influenza causes most cases of severe pneumonia in children in developing countries. S.pneumoniae colonizes the nasopharynx and can be isolated from 20-40% of healthy children (17). The incidence of pneumococcal bacteremia is relatively high among infants up to 2yrs of age (17). Studies suggest that pneumococci must invade tissues by penetrating mucosal layers to be pathogenic. Non specific host defense mechanisms including the presence of other bacteria usually in the oropharynx limit the multiplication of pneumococcus (18). Pneumococcal infection frequently follows a viral respiratory tract infection, which may produce mucosal damage, diminish the epithelial ciliary activity and depress the function of alveolar macrophages. The host response to influenza infections involves complex interplay of humoral antibody, local antibody, cell medical immunity, interferons and other host defense systems. Secretory antibodies produced in the respiratory tract are predominantly of the IgA class, and also play a role in protection against infection (19). Influenza in itself is a major cause of morbidity in the world, in addition it is important in causation of secondary bacterial pneumonia which frequently follows Influenza. In H.influenza infection the main site of cellular involvement is the mucus membrane of the respiratory tract.

Probiotics represent a potentially exciting prophylaxis and therapeutic advance for all three of these respiratory illness groups, although additional investigation must be undertaken before their role can be delineated clearly. There is evidence to suggest that indigenous microflora in the oropharynx can act competitively with pathogens, thus inhibiting their colonization and hence preventing infection. However it is true only for certain component of the indigenous flora and for certain pathogens. Lactobacilli are present in the entire respiratory tract in low levels. There is no report of the presence of Lactobacilli as part of the indigenous microflora of the respiratory tract, with the exception of Liedbeck et at (1987) who reported the impact of L.acidophillus on human oropharynx and intestinal microflora of volunteers with L.acidophillus milk. They reported values of Lactobacillus between 102-103 CFU /ml of saliva and suggested that quantification’s of microorganism from saliva reflected the number of probiotics in the oropharynx.

Several studies have demonstrated the beneficial effects of Lactic acid bacilli in enhancing non-specific immune response. Probiotic has been shown to influence immune response non specifically by enhancing phagocytosis of pathogen as well as modifying cytokine production. Studies have shown an enhanced sIgA producing plasma cells production in a dose dependent manner (20). In a study by Perdigon et al. 1991 L. casies was shown to significantly increase the amount of sIgA in response to salmonella inoculum. The increased secretion of IgA was sufficient to prevent enteric infection (21).

Given the pronounced immunomodulatory effect of Probiotics and the central importance of IgA, cytokines and mucosal barrier to respiratory tract infection, there is a strong potential for preventive role. The only conclusive way to investigate the potential benefit of probiotics in preventing diarroheal and respiratory infections or in reducing duration or severity of these illnesses would be by conducting a randomized double masked clinical trial with these clinical outcomes.

**1.2 Role of Micronutrient Zinc and Iron (in conjunction with Vit A and C)**

It is now becoming clear that a large portion of attributable risk (for increased infectious disease morbidity and mortality) to malnutrition may in fact be due largely to a few critical micronutrients. This understanding has led to considerable interest in nutritional approaches to reduce disease burden and improve child survival. There is increasing evidence for zinc and iron to be two potential candidates in this regard. Recent controlled trials in India have shown therapeutic effects of supplemented zinc on the duration and severity of acute and persistent diarrheal episodes (22-24). There has also been documentation of preventive effect of zinc supplementation on the incidence and prevalence of acute diarrhea, (25) persistent diarrhea and dysentery (26). There are strong theoretical bases for such effects as zinc deficiency impairs cell regeneration (due to its role in metalloenzyme and metabolic pathways) and impairs immunocompetence (27, 28). The size of total body zinc stores is small, which makes occurrence of deficiency more likely.

We performed a pooled analysis of all randomized controlled trials of Zinc supplementation. The data from the available 10 trials evaluating prevention effects of zinc supplementation (29), 3 trials evaluating therapeutic effects on acute diarrhea, and 4 trials in therapy of persistent diarrhea (30)have been reported. These analyses indicated that there was significant homogeneity in the results across the studies conducted in different developing countries. These studies also provide by far the best evidence for wide spread prevalence of zinc deficiency among preschool children, showing about 38-50% of developing country children as actually zinc deficient. Among the preventive trials, the pooled estimates for diarrheal incidence and prevalence indicated a 18% (95% C17 to 28%) and 25% (95% C1 12 to 37%) lower odds, respectively, in zinc supplemented compared with control children. There were also trends towards reduction of persistent diarrhea and dysentery. In the four trials that evaluated pneumonia morbidity, zinc supplemented children had 41% (95% C1 17 to 59%) lower odds for pneumonia incidence. The analysis concluded that zinc supplementation in children in developing countries results in substantial reduction in rates of diarrhea and pneumonia, the two leading causes of death in these settings.

Anemia, especially that due to Iron deficiency, is a leading cause of morbidity and mortality worldwide (31). Iron deficiency is also associated with a wide variety of abnormalities, including depression of cell mediated immune responses (32), and impairment of normal motor and cognitive development (33). Although there are no good data on preventive effects of iron supplementation either on morbidity or on mortality there have long been recommendation for iron supplementation in better developed and developing countries (34). WHO and UNICEF have included reduction of iron deficiency among their goals. Keeping in view the widespread prevalence of Iron deficiency in children especially after 6-9 months of life there is a need for Iron supplementation. The strongest documented benefit of improvement of iron status seems to be on improved behavioral and cognitive development and decrease in mortality due to severe anemia (35). Additional studies of Lozoff et al documenting developmental deficit on a long term follow up of cohort of children who were anemic in a window of time in childhood and then were replete for rest of life, suggests that even transitory deficiency in childhood may have long term consequences on behavioral and cognitive development.

**1.3 Fortified Milk as a vehicle of Intervention**

For delivery of Probiotics or Zinc and Iron (in conjunction with Vit A and C), we can either follow a supplementation path or a fortification path. The supplementation path has already been shown not to be feasible, given the experience with Iron supplementation programs. Fortifying Milk with Bifidobacillus lactis (HN019) and Oligosccharides or Zinc and Iron (and Vitamin A and C), for administration to children older then 1yr seems by far the most viable strategy to deliver these to children given the high levels of milk consumption among all age groups of children, its availability to the masses and the growing trend towards centralized collection and distribution of milk.

**2. Aims and Objectives**

In a randomized double blind controlled trial we aim to investigate the efficacy of Bifidobacillus lactis HNO19 and Oligosaccharides or Zinc and Iron (in conjunction with Vitamins A and C) in prevention of diarrhea, pneumonia and improvement in growth and development.

**2.1 Specific Aims**

To evaluate efficacy of milk fortified with HNO19 and Oligosaccharides in preventing diarrhea, pneumonia and improving growth and development in children >1yr as compared to the group receiving the same milk without additional fortification with HNO19 and Oligosaccharides.

To evaluate efficacy of milk fortified with RDA of Iron and Zinc (in conjunction with Vitamins A and C) in preventing diarrhea, pneumonia and improving growth and development in children >1yr as compared to the group receiving the same milk without additional fortification with Iron, Zinc and natural levels of vitamins A and C.

**2.2 Secondary Objective**

To evaluate relative efficacy of Bifidobacillus lactis HNO19 and Oligosaccharides vs Zinc and Iron (in conjunction with Vitamins A and C).

To evaluate the effect of fortified milks on zinc status as assessed by plasma zinc levels.

To evaluate the effect of fortified milks on iron status and anemia prevalence as assessed by hemoglobin concentration, mean corpuscular volume (MCV) and RBC distribution width (RDW), Plasma ferritin and zinc protoporphyrin and on lead and copper status.

**3. Study Design and Methodology**

**3.1 Study Population**

The study is proposed to be conducted in Sangam Vihar, N.Delhi, India. It consists of predominantly low socioeconomic resettlement population representing migrants from different rural areas of the country. The representative nature of the population makes it an ideal choice for scientific trials. The annual income of 50% of households is less than Rs.26, 000/-(~U.S $ 580/-). Sangam Vihar being an unauthorized settlement on Govt. land has no civic infrastructure (no system of electricity, water and sewage disposal). The literacy rate is 50% for men and only 6% for women. Low income, very low literacy rate among women and large family size makes the children, a vulnerable group in particular. As anthropometry reveals 5% of children have severe malnutrition and 40% have moderate malnutrition. From the previous community-based studies conducted by Sazawal et al (1996) in this area, the diarrheal incidence in 1-2yrs age group children is 6 episodes/child/year and incidence of pneumonia for the same age group is 1.23 episodes/child/year. The infant mortality rate in this population is 83 per 1000 live births. The birth rate is about 30 per 1000 population. Data on morbidity in children available with us suggests, respiratory infections including pneumonia and gastro-intestinal disorders, as the most common disease condition among children.

- 1. **Baseline Population Data Base Available**

Sangam Vihar being the field site for the Center for Micronutrient Research lends us a strategic advantage. The area has been mapped in detail and houses have been numbered. Database of household information and Socio-economic survey is available. Census of children by age are maintained and updated every 15days through population surveillance.

- 1. **Eligibility Criteria**

From the baseline census available, a list of households with children in the eligible age range will be made and distributed among the Field Monitors in charge of specified area/households. These households will then be visited; study procedures explained and consent for participation sought after evaluating inclusion and exclusion criteria.

Inclusion Criteria

- Children aged 12-36 months at enrollment
- Resident of Sangam Vihar and likely to be in the area for 1 year.
- Parents consenting to participate in the trial.

Exclusion Criteria

- Children who have been given complete therapeutic/preventive course of Iron in the last 6 months.
- Children currently as part of a different Iron/Zinc supplementation program.
- Children known to be allergic to milk.
- Children who are severely malnourished and requiring hospitalization.
- Children who are exclusively or predominantly breast feeding
- Children who have chronic or severe illness requiring hospitalization or special treatment.
  1. **Enrollment Of Eligible Children And Baseline Information:**

Eligible children will be enrolled and their enrollment forms filled. The form will include socioeconomic information, retrospective morbidity assessment of the child for 15days with special emphasis on diarrhea and pneumonia/respiratory infection, (duration, severity and no. of episodes) dietary habits and hygiene. Clinical assessment of the child will be made by the research physician and hospitalization episode recorded. Anthropometric measurement weight and length/height will be recorded. Blood will be collected to assess zinc and iron status. Global assessment of cognitive and motor development will be measured by the Bayley Scales of Infant Development, Ver. 2. Parent Child Early Relationship Assessment,(PCERA), Social Referencing (SR), Activity Assessment on a sub- sample, Toddler Behavior Assessment Questionnaire, Behavior Rating Inventory of Executive Function (BRIEF), HOME, Expressive Language questionnaire will be instituted to assess the quality of parent child relationship and physical activity levels of children. In addition, two 24 hour dietary recalls and food frequency questionnaire will be instituted. Child found anemic will be given therapeutic dose of iron and will be stratified separately.

This will allow comparison of study groups after randomization to assess the quality of equalization of baseline characteristics achieved by randomization and the need for any correction at analysis.

- 1. **Sample Size Estimation**

Assumptions used for sample size calculations are based on our current data from previous trials in this population. In age group of more than 12 months, a diarrheal incidence of 6.8 episodes per child/year, a pneumonia incidence of 1.05 episodes per child per year, mean motor development raw score of 5.51 + 2.44 and a mental development raw score 9.93 + 4.56. As the sample size for growth is least limiting it is not presented here. Also sample size for prevalence is less than that for incidence and so only incidence calculations are presented.

1. **For incidence of diarrhea, pneumonia and development**

(Assuming alpha of 0.05, two tailed test)

| Outcome | Incidence in control group | % reduction targeted | Sample of children /group with power  90% 80% | |
| --- | --- | --- | --- | --- |
| Episodes of diarrhoea | 0.019 (6.8 episodes/child yr.) | 10 | 300 | 225 |
|  |  | 15 | 131 | 98 |
| Episodes of pneumonia | 0.003 (1.05 episodes/child yr.) | 25 | 276 | 208 |
| Motor development | 5.51 + 2.44 | 30 | 235 | 176 |
| Mental development | 9.93 + 4.56. | 30 | 235 | 176 |

1. **Sample size for duration of episodes**

With a sample size of 531 episodes per group, an exponential maximum likelihood test of equality of survival curves for duration of episodes with a 0.05 two‑sided significance level will have 90% power to detect a reduction of 20% (control group exponential parameter 0.35, hazard ratio of 0.8); this assumes an accrual period of 30, a maximum follow up time of 365 days, and a common exponential dropout rate of 10%. To detect a reduction of 10% (control group parameter 0.3, hazard 0.8) the sample size required is 1041 episodes - approximately 200 children after accounting for attrition.

C) Sample size for multivariate analysis for developmental assessments

The specific sample size necessary to achieve adequate statistical power (e.g., .80) in a regression analysis depends largely on the effect sizes of the variables and the numbers of variables included in testing the hypotheses, so an overall estimate of statistical power is not possible. A series of power analyses was undertaken that assumed modest effect sizes (e.g. 10% explained variance) and that included various independent variables typical of the proposed hypotheses. These power analyses were done using a general linear model as estimated using multiple regression (Bornstein & Cohen, 1988). For example, suppose that for 200 children development was regressed on intervention group and five quantitative variables thought to mediate the effects were included (resulting in a total of 9 variables). Assuming a modest effect size, the same for the combined effects of iron and zinc for the mediating variables (i.e., R^2^_change_ = .10), and no main effects for the mediating variables, the power to detect the overall R^2^ of .20 equals .94, the power to detect the effect of HNO19 or Zinc/Iron equals .89, and the power to detect the combined main effects equals .85.

Given the sample size estimations shown above a sample size of 350 per group is proposed so that after accounting for attrition and loss of follow up due to temporary non availability we would have a person time available equivalent to 300 child years per group. This sample size would be adequate for all clinically meaningful differences.

- 1. **Randomization and Double Blinding**

Randomization schedule will be drawn using randomization by permuted blocks of fixed length, which is appropriate for double blind studies. This will be stratified by presence of severe anemia at baseline. Enrolled children will be given a study serial number, which will be used to allocate them an intervention code (A through D) from the randomization chart. The intervention packets will be labeled by four codes A through D; all the packets will be identical in appearance and the master list of identification of groups A-D will not be available to the investigators till the end of the study, (this identification will be known only to the company and the monitoring committee). The randomization chart, which will be drawn in advance, will give the designated code for each child ID number. Once a child is enrolled based on his/her child ID number he or she will be allocated to receive one of the four alphabetical labels and hence preparations. The alphabetical allocation will only be known to the pharmacy assistant who will have the randomization chart, he will label the packet with specific information of the child. In the field, the milk packets will be sent with the child name and the number on them.

## **3.7 Intervention Groups**

There will be two study groups with their own control groups. One study group would receive 96 g of Milk A (Growing up Milk Powder) fortified with Bifidobacillus lactis HNO19 and Oligosaccharides (henceforth referred to as HNO19 group) in three divided doses reconstituted with 600 ml water. The control group would receive 96 mg Milk A (GUMP-Growing up Milk Powder) without additional fortification with HNO19 and Oligosccharides (henceforth referred to as the GUMP group).

The second study group would receive 96 mg of Milk B (Budgie formula) fortified with 100% of the US RDA for bioavailable Zinc, Iron, Vitamin A and Vitamin C (henceforth referred to as Zinc/Iron group) in three divided doses reconstituted with 600 ml water. The control group would receive 96 mg Milk B without additional fortification of Zinc or Iron and natural levels of Vitamins A and C (henceforth referred to as Control group).

### **3.8 Intervention Delivery**

Airtight packets marked with randomized codes will be prepared by the company in advance and maintained at the central office. The study pharmacy assistant will label them with the child ID and name before sending them to the field. Each packet will contain 21 x 32 g packets of milk powder meant for a week. The field monitor will deliver the Milk packet and demonstrate the reconstitution procedure and educate the mother of the dosage and frequency of administration 32 g milk reconstituted to 200 ml, given three times a day. Children usually adapt to three-meal schedule at the end of 1 year and personal idiosyncrasies become more overt at this stage. The field monitor will advise and explain the mother regarding the futility of forced dietary consumption. The weekly visit will also be utilized to assess compliance.

### **3.9 Concurrent Medications**

No restriction will be exercised regarding medical treatment, which will be given to the patient based on the child’s clinical condition. No restriction on antibiotic or other medication will be posed. The research physician being blind to the trial cannot yield to bias. Management of diarrhea and pneumonia will be undertaken in accordance to the WHO recommendations. However, any medication for diarrhea, pneumonia or other illnesses taken by children will be recorded.

#### **4. Assessment of Outcomes**

##### **4.1 Delivery of Milk and Assessment of Compliance**

After enrollment the Monitor will visit each child at home once a week. S/he will deliver the milk for the next week and will collect the Compliance data of the previous week. Intake by day will be recorded at each of the visit on a prescribed form, for the previous 7days. In the event of the child’s absence, a repeat visit will be made the next day.

- 1. **Follow up - Morbidity Assessment**

Specially trained Health Assessment Monitor will make his/her visit twice a week and will record information on hospitalization, diarrhea, acute respiratory infection / pneumonia and fever on that day and the preceding 3-4 days on a standard form.

Diarrhea will be defined as 3 or more loose, liquid, or watery stools or at least one bloody stool in 24 hrs. An episode is considered resolved on the last day of diarrhea if there is at least 3 intervening diarrhea free days. Feeding pattern during illness or diarrhea will be duly recorded.

Acute lower respiratory tract infection information will be collected on the basis of fever, cough, nasal discharge and otitis. If cough was present on the day of visit, child is examined to determine the rate of respiration and the presence of chest indrawing. ALRI is defined as the presence of cough and respiratory rate >50/min with or without chest indrawing (Selwyn, 1990). Diagnosis of pneumonia will be made on clinical ground by the research physician. A new episode will be considered if there is an interval of 7 or more ALRI free days.

- 1. **Follow-up – Growth, Development, Physical Activity and Dietary Assessment**

The weight, height/length will be taken at 6 and 12 months of study. Global assessment of cognitive and motor development will be measured by the Bayley Scales of Infant Development, Ver.2 at 6 months and at 12 months of enrollment. After each administration of the Bayley Scales, the examiner will complete the Behavior Rating Scale (BRS) from the Bayley Scales, based on the child’s response to the structure of the testing situation. These scales enable the examiner to rate the children on behaviors that have been shown to be sensitive to nutritional factors. Parent Child Early Relationship Assessment (PCERA), Toddler Behavior Assessment Questionnaire, Behavior Rating Inventory of Executive Function (BRIEF), Expressive Language questionnaire, and Social Referencing (SR) will be assessed at mid-study. Activity Assessment on a sub- sample, HOME, Parental Stress Index (PSI) and Behavior and Social Interactions (BASIS) will be administered at 12 months to assess the quality of parent child relation and physical activity levels of children. Dietary intake using 24 hour dietary recall and food frequency questionnaire will be recoded 2 times at Mid-study and 2 times at End-study.

- 1. **Blood sampling At End Study**

Blood samples will be collected using standard methods to evaluate zinc, iron, copper and lead status.

1. Ethical Considerations

Before recruitment and enrollment into the study, each prospective candidate will be given a full explanation of the study and informed written consent sought and recorded. The study protocol will be approved by the IRB of Hopkins University and the ethics committee at Annamalai University.

###### **6. Staff Training and Supervision**

The center for Micronutrient Research has specially trained technical manpower to carry out trial of this nature. For the trial the fresh field staff will be recruited and given orientation of 2 weeks prior to the start of the trial. This will include the explanation of the final tools of investigation (questionnaires, treatment protocols), detail regarding the protocol. We have a standard practice of attaining reliability for all measurements. How ever as most of these assessments are being undertaken presently for older children this project will have a short startup time. A standard manual of operations will be designed for this study during startup period.

1. Data Management

The investigators at Center for Micronutrient Research have long experience of establishing data management facilities at field sites. The system proposed has been successfully used for the recent Zinc supplementation trials. Oracle and Visual Basic data management software will be used. This has screen designs similar to the forms and has built in range and logical checks. A separate database for tracking the data management is used, such that each visit to a child is a piece of data. On a day to day basis data is entered. The person entering the data also completes the tracking system. The files from individual records are themselves stored in duplicate, and uploaded into the main system on a twice-weekly basis where a child-specific database is constructed. Each day’s field data forms are checked on the same day and handed over to the computing office. The main computer data is also used for quality control management and supervision by tracking of measurements, as well as errors, made by field staff.

1. Data and Safety Monitoring Committee

The purpose of the Data and Safety Monitoring Committee is to provide external, objective advice to the investigators regarding the safety and efficacy of the interventions being evaluated. This committee will be responsible for reviewing the data from the study on a regular basis, summarizing their conclusions and advice in a written set of minutes, and communicating this information to the investigators. They have specific responsibility to determine if there are problems relating to the safety of the interventions to the degree that the trial should be stopped and to determine if the study should be stopped early because the benefit of the interventions has been proven. The Data and Safety Monitoring Committee will meet on approximately a 6 monthly basis. Membership on the Data Safety and Monitoring Committee (DSMC) is limited to persons external to the investigative team and the funding agencies. Senior members of the investigative team will serve as resources to the DSMC. Donor agency representatives may participate in the open sessions of the DSMC.

Voting Members

To be named: Senior Child Health Specialist

To be named: Bio-statistician

To be named: Public Health Expert

To be named: Nutritionist

Investigator Resource Team

Sunil Sazawal, Harsharan Gill, Venugopal P. Menon, Lesley Stevenson

**9. Data Analysis**

Data analysis activities will be conducted at regular intervals throughout the study period corresponding to the dates of the Data and Safety Monitoring Committee (DSMC) meetings. Approximately one month before a scheduled DSMC meeting, the data set will be frozen for analysis. Data collection and management will continue, but the analysis for that meeting of the DSMC will be based on the frozen data set. This data analysis will be performed by DSMC statistician who will have the code. He will be handed over data files without study group code.

In the final data analysis first exploratory data analytic techniques will be used to examine the distributional properties of the full range of study variables. Over and above what was done during the data management phase, additional checks for range, consistency and validity will be conducted. If necessary, some variables may be mathematically transformed so their distributional properties are more consistent with standard statistical approaches to estimation and hypothesis testing. During this phase, compound variables will be created as well. These will include summary measures of socioeconomic status, calculation of episodes of morbidities and others.

The second phase of the analysis will focus on conducting baseline comparisons of the four treatment groups in order to understand if there are imbalances on potentially important confounding variables that occurred despite the randomization process. This will include comparisons of household demographic (age and sex) and socioeconomic characteristics (caste, educational level of the mother and father, occupation of the head of household, land and animal ownership, etc.), baseline prevalence rates of the various morbidities as assessed during the enrollment interview with the parents, and baseline measures of height, weight, height‑for‑age, weight‑for‑height. Both measures of central tendency (mean and median) and full distributions of these variables will be compared between the two treatment groups. Variables that are identified to be imbalanced among the treatment groups will be adjusted for in the outcomes analysis using a variety of statistical techniques such as linear, logistic, Poisson, and Cox proportional hazards regression models, as well as more simple stratified approaches using direct adjustment techniques.

The primary analysis will then focus on the specific aims of the study.

**10. Analytical Strategy**

**10.1** For the purpose of description of comparisons following abbreviation of groups will be used:

HNO19 group - Children fed Milk A (growing up milk powder) fortified with B. Lactis HNO19 and milk derived oligosaccharides

GUMP group - Children fed Milk A (GUMP), without addition of HNO19 and oligosaccharaides

Zinc/Iron group - Children fed Milk B (Budgie formula), with additional fortification of zinc and iron to provide RDA of both.

Control group - Children fed Milk B (Budgie formula), without fortification.

(Note: The indicative formulations of the four test milks are attached to this proposal. Some nutrient levels require confirmation).

The analytical strategy will be to make two primary comparisons and two secondary comparisons. The two primary comparisons will include a) Comparing HNO19 group with GUMP group which will give impact of addition of B.lactis and Oligosaccharides to GUMP milk formula, b) comparing Zinc/Iron group and control group which will provide impact of zinc and iron fortification of Budgie formula. The two secondary comparisons will consist of a) comparing HNO19 group with control group, which will provide combined impact of improved milk formulation compared to Budgie formulation, b) comparison of impact of zinc/iron with impact of HNO19, which will provide relative impact of two intervention. For all these comparisons three sets of analysis will be performed.

Analysis I: Effect on the duration and severity of the diarrheal episodes (episode based analysis).

Analysis II: Effect on the incidence and prevalence of diarrhoea and pneumonia during 1-year follow-up

Analysis III: Effect on growth and development during 1-year follow-up.

Analysis IV: Effect on physical activity levels during 1-year follow-up.

Analysis V: Effect on iron and zinc status and anemia prevalence during 1-year follow-up.

Analysis VI: Effect on blood lead levels and plasma copper status during 1-year follow-up.

This analysis will use two approaches to estimate the impact of intervention on child morbidity. First, we will conduct a person‑years analysis where the amount of person‑time contributed by each study subject will be calculated. Morbidity rates will then be calculated for all four groups on a per child‑years basis. These incidence densities will be compared using the relative risk and appropriate confidence intervals will be placed on the relative risk and the crude mortality rates (60). The second approach will be use multivariate methods described subsequently.

**10.2. Effect on duration**

In the first analysis the duration of episodes will be analysed using Cox survival regression models with a time dependant covariate (PHREG procedure in SAS 8.0 using "*exact option*" for handling of ties) (164,165). The duration (D) of episode with be modelled as dependant variable, while intervention group will be entered as independent variable. Multivariate analyses with inclusion of covariates and interaction terms in these models will also performed. For the covariates that are found to be significantly associated with duration, a stratified Cox regression will be used (166,167). In the second analysis the proportion of episodes lasting more than 7 days will be evaluated by logistic regression models with duration (>7 days/7 days) as the dependant variable and intervention (yes/no) and potential covariates, and interaction terms as independent variables. The mean number of watery stools and diarrheal stools will be evaluated by Poisson regression model.

**10.3 Effect on incidence and prevalence of diarrhoea and pneumonia**:

Relative rates for the effect of addition of HNO19 and oligosaccharides to GUMP and addition of Zinc and Iron to budgie milk formula will be estimated using Poisson regression models. The simplest model, y= log (# episodes) and x= group allocation (0 for control, 1 for intervention), with log transformed actual follow up as offset term, provides a rate ratio of the effect of zinc supplementation on incidence. Odds ratio for prevalence will be estimated using marginal logistic regression models, with the events/trials option (SAS 8.0 Proc Logistic (168), y = logit ( days of RR more than cut off / actual follow up), and x= group allocation (0,1). In order to take the over dispersion due to within child correlation into account, quasi-likelihood estimates will be used (169). Residual deviance and its degrees of freedom for both regressions will be used to estimate Φ=(residual deviance/ degrees of freedom). Rate ratios and odds ratios will be estimated as exp (β), while corrected standard errors were obtained by multiplying estimates from the model with Φ. The corrected standard error will then be used to estimate the 95% CI for β.

In an alternative analysis, each month of observation will be treated as a single observation and thus each child will potentially have 12 observations. Covariates will be reset for each interval. In this analysis, incidence of pneumonia or persistent diarrhoea will be treated as a bivariate variable. GEE methods will then be used for modelling incidence. Similarly days of diarrhoea in month will be modelled using logistic method within GEE for prevalence.

**10.4. Effect on growth**:

The weights and lengths will be used to calculate the growth velocity (Weight velocity = g/month and g/6 months; Height velocity = cm/month and cm/6months), HAZ, WAZ and WHZ (z-scores for height for age, weight for age and weight for height respectively) and change in z-scores during the study period will be calculated. Difference in means between intervention and control groups will be evaluated using non-parametric tests. Analysis will be performed using SPSSPC+, Epi-Info, and SAS. For anthropometry Z values will be calculated by CDC software and appended to the data set.

For the growth velocity, two stage random effects models appropriate for the longitudinal data will be used. In the first stage, the correlation matrix will be estimated and then that will be used to fit a random effects model. For the weight or height increments both random and fixed effects will be modelled. Proc Mixed (SAS 8.0) will be used for random effect modelling. Centering of variables will be used if there is colinearity. If growth effect and morbidity effect are both found, the role of morbidity as a mediator of growth effect will be assessed.

**10.5 Effect on development**:

We will use two types of comparisons of developmental assessment, firstly we will compare the intervention and control groups for difference in mean raw scores (Bayley mental, motor, and behaviour scores) between baseline and 6 months and 1 year assessment. In these analyses we will use multivariate regression analyses with gender and socio-economic status as covariates and supplementation group as the primary independent variable. Because children’s age, family environment and temperament may also influence behaviour and developmental scores, they will be entered into the analysis prior to supplementation group and then interacted with supplementation group to examine whether the impact of supplementation is exacerbated or mitigated by current age, family environment, or temperament. In addition to performing a raw score analysis, the results from Bayley Scores will be converted into z-scores using the international reference that will permit group comparisons irrespective of age of assessments. Use of an international reference in this case is valid as the comparisons are being still made within the treatment groups.

In the second analysis we will use hierarchical linear modelling (HLM) to examine the effects of supplementation group and environmental variables on changes in children’s development during one year of study. HLM is a 2-stage procedure for estimating intra individual change and correlates of change (Bryk and Raudenbush, 1992; Burchinal et al., 1994; Burchinal et al., 1997). In the first stage (the within-subject stage), growth curves will be individually formulated for each child. They will be evaluated for linear, square, or cubic forms depending on within-child patterns of change. In the second stage (the between-subjects stage), group and environmental variables will be used to examine the intercept and slope of the individual growth curves. The relations between the multiple measures of development and weight and both time varying (i.e., age) and time-invariant covariates (i.e., supplementation group) will be examined simultaneously.

In this study, at Level 1, each child’s developmental growth pattern will be modelled as a function of age, represented by the following model Y_ti_ = π_0i_ + π_1i_a_ti_ (linear) + π_2i_a^2^_ti_(square) + π_2i_a^3^_ti_ (cubic) +.........e_ti_ where Y_ti_ is the outcome variable for child I measured at time t; π_0i_ is the mean outcome for child I; π_1i_a_ti_ is the average linear developmental growth rate in Y for child I over time and a_ti_ is the age of child I at time t; π_2i_ and π_3i_ are the square and cubic rates of acceleration; and e is the random error term which is assumed to be normally distributed with a mean of zero and constant variance. At Level 1, the error may be child specific or as in this study could depend on errors in measurement.

The Level 2 model for the intercept is π_0i_= β_0i_ + β_01_ (predictor 1)_I_ + β_02_ (predictor 2)I .........+ e_0i_ where the level 1 intercept (π_0i_) is viewed as dependent on the Level 2 intercept (β_0i_) and the measured characteristic of the environment, plus the random effect. The Level 2 model for the linear slope is π_1i_ = β_10_ + β_11_ (predictor 1)_I_ + β_12_ (predictor 2)_I.._. + e_1i_. The Level 1 slope for each child is dependent on the level 2 intercept, group, environment, and random error.

The dependent variables will be measurements in development (raw scores of motor and cognitive development) collected at multiple age points. We will examine three additive models. The first model identifies supplementation group. The second model examined any additional variance accounted for by child factors, including health, temperament, and gender. In the third additive model, we will examine the role of environmental characteristics (parental nurturance, maternal depression, and socio-demographic characteristics). To examine if the relationship between developmental growth and the child and family variables differed by the children’s early growth history, we will include interaction terms (e.g., group X child health) in the second and third models.

**References**

1 Investigating in Health. World Bank Development Report. New York : OUP, 1993.

2 Black RE, Mosley WH, Chen LC et al. Diarrheal diseases and child morbidity and mortality. Child survival strategies for research Popul & Dev Rev 1984; Suppl to 10.

3 Gomaa A et al. Impact of the National control of diarrhoeal diseases project of infant and child mortality in Dakahlia. Egypt. Lancet 1988; ii:145-8.

4 Rehman MM, Aziz KMS et al. Diarrhoeal mortality in two Bangladeshi villages with and without community based oral rehydration therapy. Lancet 1979; ii:809-12.

5 Tekce B. Oral rehydration therapy : an assessment of mortality effects in rural Egypt. Study Family Plan 1982; 13:315-27.

6 Van der Waaij.D, de Vries JMB, Lekkerkerk van der Wees JEC. Colonization resistance of mice during systemic antibiotic treatments. J Hyg 1972; 70:605-9.

7 Sanders ME. Considerations for Use of Probiotic Bacteria to Modulate Human Health. J Nutr 2000; 130:384S-90S.

8 Guamer F, Schaafsma GJ. Probiotics. Int J Food Microbiol 1998; 39:237-8.

9 Rolfe RD. The Role of Probiotic Cultures in the Control of Gastrointestinal Health. J Nutr 2000; 130:396S-402S.

10 Gut 2000; 42:2-7.

11 Lancet 2000; 244:1046-50.

12 Lancet 2000; 347:1017-20.

13 Arunachalam et al. Eur J Clinical Nutrition 2000; In Press.

1. Gill et al. 2000.
2. Gill et al. British Journal of Nutrition 2000; In Press.

16 Shu et al. Microbiology and Immunology 2000; In Press.

17 Musher DM. Pneumococcal Infections. In: Fauci AS et al., editor. Harrison's principles of internal medicine. The McGraw-Hill Companies Inc., 1998: 869-75.

18 Behrman RE, Vaughan VC. Pneumococcal Infections. In: Nelson WE, editor. Nelson Text Book of Pediatrics. W.B.Saunders Company, 1983: 638-41.

19 Madoff LC, Kasper DL. Introduction to Infectious Diseases : Host-Parasite Interaction. In: Fauci AS et al., editor. Harrison's principles of internal medicine. The McGraw-Hill Companies, 1998: 749-53.

20 Perdigon D, Alvarez S, Rachid M et al. Immune system stimulation by probiotics. J Dairy Sci 1995; 78:1597-606.

21 Perdigon D, Alvarez S, Pesce de Ruiz Holgado A. Immunoadjuvant activity of oral Lactobacillus casei : influence of dose on the secretory immune response and protective capacity in intestinal infections. J Dairy Res 1991; 58:485-96.

22 Sachdev HPS, Mittal NK, Mittal SK et al. A controlled trial on utility of oral Zinc supplementation i acute dehydrating diarrhea in infants. J Pediatr Gastr Nutr 1988; 7:877-81.

23 Sachdev HPS, Mittal NK, Yadav HS et al. Oral Zinc supplementation in Persistent diarrhoea in infants. Ann Trop Paediatr 1990; 10:63-9.

24 Sazawal S, Black RE, Bhan MK et al. Effect of zinc supplementation during acute diarrhea on duration and severity of the episode - A community based, double-blind controlled trial. N Eng J Med 1995; 333:839-44.

1. Sazawal S, Black RE, Bhan MK, Jalla S, Sinha A, and Bhandari N. Efficacy of zinc supplementation in reducing the incidence and prevalence of acute diarrhea - A community-based, double-blind, controlled trial. 9999.
2. Sazawal S, Black RE, Bhan MK et al. Zinc supplementation reduces the incidence of persistent diarrhea and dysentery among low socioeconomic children in India. J Nutr 1996; 126:443-50.

27 Chandra RK et al. Single nutrient deficiency and cell mediated immune response. I.Zinc. Amer J Clin Nutr 1980; 33:736-8.

28 Stabile A et al. Immunodefeciency and Plasma Zinc levels in children with Down's Syndrome : A long term follow up of oral zinc supplementation. Clin Immunol and Immnopathol 1991; 58:207-16.

29 Bhutta ZA, Black RE, Brown KH, Meeks Gardner J, Gore S, Hidayat A, Khatun F, Martorell R, Ninh NX, Penny ME, Rosado JL, Roy SK, Ruel M, Sazawal S, and Shankar A. A Prevention of diarrhea and pneumonia by zinc supplementation i children in developing countries. 1998.

30 Bhutta ZA, Black RE, Brown KH, Meeks Gardner J, Gore S, Hidayat A, Khatun F, Martorell R, Ninh NX, Penny ME, Rosado JL, Roy SK, Ruel M, Sazawal S, and Shankar A. Therapeutic Effects of Oral Zinc in Acute and Persistent Diarrhea in Children in Developing Countries : Pooled Analysis of Randomized Controlled Trials. 9999.

31 Ad Hoc Committee on Health Research Relating to Future Intervention Options. Investing in health research and development. TDR/GEN/96.1. 1996. Geneva, WHO.

1. Thibault H, Galan P, Selz F et al. The immune response in iron-deficient young children: effect of iron supplementation on cell-mediated immunity. Eur J Pediatr 1993; 152:120-4.

33 Lozoff B, Jimenez E, Wolf AW. Long-term developmental outcome of infants with iron deficiency. N Engl J Med 1991; 325:687-94.

34 Committee on Nutrition. Relationship between iron status and incidence of infection in infancy. Pediatrics 1978; 62:246-50.

35 Stoltzfus RJ, Dreyfuss ML. Guidelines for the Use of Iron Supplements to Prevent and Treat Iron Deficiency Anemia. International Life Sciences Institute, 1998.

**Product Name:** MILK A – indicative (HN019 Group)

| **NUTRITION INFORMATION** | | | | |
| --- | --- | --- | --- | --- |
|  | per 100 g powder | | per serve (32 g powder) | |
| Energy | 1980 | kJ | 630 | kJ |
|  | 470 | kcal | 150 | kcal |
| Protein | 21 | g | 6.7 | g |
| Carbohydrate | 52 | g | 16.7 | g |
| Oligosaccharides | 3.5 | g | 1.1 | g |
| Fat | 20 | g | 6.4 | g |
| Linoleic Acid | 4.2 | g | 1.3 | g |
| Linolenic Acid | 0.49 | g | 0.16 | g |
| DHA | 35 | mg | 11.2 | mg |
|  |  |  |  |  |
| Vitamin A | 400 | μg^*^ | 130 | μg^*^ |
|  | 1330 | IU | 430 | IU |
| Vitamin D | 5.2 | μg | 1.7 | μg |
|  | 210 | IU | 67 | IU |
| Vitamin E | 6.2 | mg^**^ | 2.0 | mg^**^ |
|  | 6.2 | IU | 2.0 | IU |
| Vitamin K | 9.8 | μg | 3.1 | μg |
| Vitamin C | 50 | mg | 16 | mg |
| Thiamin | 0.5 | mg | 0.2 | mg |
| Riboflavin | 1.0 | mg | 0.3 | mg |
| Niacin | 6.2 | mg | 2.0 | mg |
| Vitamin B_6_ | 0.5 | mg | 0.2 | mg |
| Pantothenic Acid | 1.0 | mg | 0.3 | mg |
| Biotin | 13 | μg | 4.1 | μg |
| Folate | 78 | μg | 25 | μg |
| Vitamin B_12_ | 1.3 | μg | 0.4 | μg |
| Choline | 90 | mg | 29 | mg |
| Calcium | 720 | mg | 230 | mg |
| Phosphorus | 515 | mg | 165 | mg |
| Iron | 8.7 | mg | 2.8 | mg |
| Magnesium | 65 | mg | 21 | mg |
| Zinc | 4.0 | mg | 1.3 | mg |
| Iodine | 40 | μg | 13 | μg |
| Sodium | 255 | mg | 82 | mg |
| Potassium | 750 | mg | 240 | mg |
| Chloride | 630 | mg | 200 | mg |
| DR10 Bifidus min 10^8^ CFU/100 g  ^*^ μg retinol equivalents  ^**^mg α - tocopherol equivalents | | | | |

**Ingredients:** Skim milk, Corn syrup solids, Cream, Sucrose, Vegetable oils (soya and sunflower), Lactose, Fish oil, DR-10 Bifidus, Lecithin, Vanillin, **Vitamins:** Vitamin A, Vitamin D_3_, Vitamin E, Thiamin hydrochloride, Pyridoxine hydrochloride, Vitamin C, Folate, Niacinamide, **Minerals:** Ferrous sulphate, Zinc sulphate.

**Product Name:** MILK A – indicative control (GUMP Group)

| **NUTRITION INFORMATION** | | | | |
| --- | --- | --- | --- | --- |
|  | per 100 g powder | | per serve (32 g powder) | |
| Energy | 1980 | kJ | 630 | kJ |
|  | 470 | kcal | 150 | kcal |
| Protein | 21 | g | 6.7 | g |
| Carbohydrate | 52 | g | 16.7 | g |
| Oligosaccharides | 0 | g | 0 | g |
| Fat | 20 | g | 6.4 | g |
| Linoleic Acid | 4.2 | g | 1.3 | g |
| Linolenic Acid | 0.49 | g | 0.16 | g |
| DHA | 35 | mg | 11.2 | mg |
|  |  |  |  |  |
| Vitamin A | 400 | μg^*^ | 130 | μg^*^ |
|  | 1330 | IU | 430 | IU |
| Vitamin D | 5.2 | μg | 1.7 | μg |
|  | 210 | IU | 67 | IU |
| Vitamin E | 6.2 | mg^**^ | 2.0 | mg^**^ |
|  | 6.2 | IU | 2.0 | IU |
| Vitamin K | 9.8 | μg | 3.1 | μg |
| Vitamin C | 50 | mg | 16 | mg |
| Thiamin | 0.5 | mg | 0.2 | mg |
| Riboflavin | 1.0 | mg | 0.3 | mg |
| Niacin | 6.2 | mg | 2.0 | mg |
| Vitamin B_6_ | 0.5 | mg | 0.2 | mg |
| Pantothenic Acid | 1.0 | mg | 0.3 | mg |
| Biotin | 13 | μg | 4.1 | μg |
| Folate | 78 | μg | 25 | μg |
| Vitamin B_12_ | 1.3 | μg | 0.4 | μg |
| Choline | 90 | mg | 29 | mg |
| Calcium | 720 | mg | 230 | mg |
| Phosphorus | 515 | mg | 165 | mg |
| Iron | 8.7 | mg | 2.8 | mg |
| Magnesium | 65 | mg | 21 | mg |
| Zinc | 3.3? | mg | 1.1? | mg |
| Iodine | 40 | μg | 13 | μg |
| Sodium | 255 | mg | 82 | mg |
| Potassium | 750 | mg | 240 | mg |
| Chloride | 630 | mg | 200 | mg |
| ^*^ μg retinol equivalents  ^**^mg α - tocopherol equivalents | | | | |

**Ingredients:** Skim milk, Corn syrup solids, Cream, Sucrose, Vegetable oils (soya and sunflower), Lactose, Fish oil, DR-10 Bifidus, Lecithin, Vanillin, **Vitamins:** Vitamin A, Vitamin D_3_, Vitamin E, Thiamin hydrochloride, Pyridoxine hydrochloride, Vitamin C, Folate, Niacinamide, **Minerals:** Ferrous sulphate.

**Product Name:** MILK B – indicative (Zinc/Iron Group)

| **NUTRITION INFORMATION** | | | | |  | | |
| --- | --- | --- | --- | --- | --- | --- | --- |
|  | per 100 g powder | | per serve (32 g powder) | |  | | |
| Protein | 21 | g | 6.7 | g |  | | |
| Carbohydrate | 52 | g | 16.7 | g |  | | |
| Fat | 20 | g | 6.4 | g |  | | |
|  |  |  |  |  |  | | |
| Linoleic acid | 2.4 | g | 0.075 | g |  | | |
|  |  |  |  |  |  | | |
| Vitamin A | 400 | μg^*^ | 130 | μg^*^ |  | | |
|  | 1330 | IU | 540 | IU |  | | |
| Beta-carotene | 120 | μg | 38 |  |  | | |
| Vitamin D | 5.2 | μg | 1.7 | μg |  | | |
|  | 210 | IU | 67 | IU |  | | |
| Vitamin E | 6.2 | mg^**^ | 2.0 | mg^**^ |  | | |
|  | 6.2 | IU | 2.0 | IU |  | | |
| Vitamin C | 50 | mg | 16 | mg |  | | |
| Thiamin | 0.5 | mg | 0.2 | mg |  | | |
| Riboflavin | 1.3 | mg | 0.4 | mg |  | | |
| Niacin | 6.2 | mg | 2.0 | mg |  | | |
| Vitamin B_6_ | 0.5 | mg | 0.2 | mg |  | | |
| Pantothenic Acid | 3.0 | mg | 1.0 | mg |  | | |
| Biotin | 20 | μg | 6.4 | μg |  | | |
| Folate | 78 | μg | 25 | μg |  | | |
| Vitamin B_12_ | 1.5 | μg | 0.48 | μg |  | | |
| Choline | 90 | mg | 29 | mg |  | | |
| Calcium | 720 | mg | 230 | mg |  | | |
| Phosphorus | 515 | mg | 165 | mg |  | | |
| Iron | 10 | mg | 3.2 | mg |  | | |
| Magnesium | 65 | mg | 21 | mg |  | | |
| Zinc | 10 | mg | 3.2 | mg |  | | |
| Selenium | 3-4? | μg | 1-1.3 | μg |  | | |
| Copper | 0.3-0.5 | mg | 0.1-0.2 | mg |  | | |
| Iodine | 40 | μg | 13 | μg |  | | |
| Sodium | 255 | mg | 82 | mg |  | | |
| Potassium | 960 | mg | 310 | mg |  | | |
| Chloride | 600 | mg | 190 | mg |  | | |
| ^*^ μg retinol equivalents  ^**^mg α - tocopherol equivalents | | | | |  |  |  |
|  | | |  | |  |  |  |

**Ingredients:** Skim milk, **ADDED CARBOHYDRATE**, Cream, Sucrose, lactose, Corn oil, Lecithin, Vanillin, **Vitamins:** Vitamin A, Vitamin D_3_, Vitamin E, Thiamin hydrochloride, Pyridoxine hydrochloride, Vitamin C, Folate, Niacinamide, **Minerals:** Ferrous sulphate, Zinc sulphate, Copper sulphate?

**Product Name:** MILK B – indicative control (Control Group)

| **NUTRITION INFORMATION** | | | | |  | | |
| --- | --- | --- | --- | --- | --- | --- | --- |
|  | per 100 g powder | | per serve (32 g powder) | |  | | |
| Protein | 21 | g | 6.7 | g |  | | |
| Carbohydrate | 52 | g | 16.7 | g |  | | |
| Fat | 20 | g | 6.4 | g |  | | |
|  |  |  |  |  |  | | |
| Linoleic acid | 2.4 | g | 0.075 | g |  | | |
|  |  |  |  |  |  | | |
| Vitamin A | 220? | μg^*^ | 70 | μg^*^ |  | | |
|  | 730 | IU | 230 | IU |  | | |
| Beta-carotene | ? | μg | ? | μg |  | | |
| Vitamin D | 5.2 | μg | 1.7 | μg |  | | |
|  | 210 | IU | 67 | IU |  | | |
| Vitamin E | ND? | mg^**^ |  | mg^**^ |  | | |
|  | ND? | IU |  | IU |  | | |
| Vitamin C | 50 | mg | 16 | mg |  | | |
| Thiamin | 0.5 | mg | 0.2 | mg |  | | |
| Riboflavin | 1.3 | mg | 0.4 | mg |  | | |
| Niacin | 6.2 | mg | 2.0 | mg |  | | |
| Vitamin B_6_ | 0.5 | mg | 0.2 | mg |  | | |
| Pantothenic Acid | 3.0 | mg | 1.0 | mg |  | | |
| Biotin | 20 | μg | 6.4 | μg |  | | |
| Folate | 78 | μg | 25 | μg |  | | |
| Vitamin B_12_ | 1.5 | μg | 0.48 | μg |  | | |
| Choline | 90 | mg | 29 | mg |  | | |
| Calcium | 720 | mg | 230 | mg |  | | |
| Phosphorus | 515 | mg | 165 | mg |  | | |
| Iron | 0.2? | mg | 0.06? | mg |  | | |
| Magnesium | 65 | mg | 21 | mg |  | | |
| Zinc | 3.3? | mg | 1.1? | mg |  | | |
| Selenium | 3-4? | μg | 1-1.3? | μg |  | | |
| Copper | ND? | mg | ND? | mg |  | | |
| Iodine | 40 | μg | 13 | μg |  | | |
| Sodium | 255 | mg | 82 | mg |  | | |
| Potassium | 960 | mg | 310 | mg |  | | |
| Chloride | 600 | mg | 190 | mg |  | | |
| ^*^ μg retinol equivalents  ^**^mg α - tocopherol equivalents | | | | |  |  |  |

**Ingredients:** Skim milk, **ADDED CARBOHYDRATE**, Cream, Sucrose, lactose, Corn oil, Lecithin, Vanillin, **Vitamins:** Vitamin D_3_, Thiamin hydrochloride, Pyridoxine hydrochloride, Vitamin C, Folate, Niacinamide.

**restart#**
